# Why is school leadership key to transforming education? Structural and cultural assumptions for quality education in diverse contexts

**DOI:** 10.1007/s11125-022-09625-6

**Published:** 2022-10-31

**Authors:** Monica Mincu

**Affiliations:** 1 Department of Philosophy and Educational Sciences, University of Turin, Palazzo Nuovo, Via Sant’Ottavio, 20, 10124 Torino, TO Italy; 2 Institute of Education, UCL, Centre for Educational Leadership, London, United Kingdom

**Keywords:** School leadership, Governance, Transformation of education, School effectiveness, Cultural contexts

## Abstract

Failing to recognize the role of leaders in quality and equitable schooling is unfortunate and must be redressed. Leadership is fundamentally about organized agency and collective vision, not managerialism, since it is an organizational quality, not merely a positionality attribute. Most important, if change is to be systemic and transformative, it cannot occur uniquely at the individual teachers’ level. School organization is fundamental to circulating and consolidating new innovative actions, cognitive schemes, and behaviors in coherent collective practices. This article engages with the relevance of governance patterns, school organization, and wider cultural and pedagogical factors that shape various leadership configurations. It formulates several assumptions that clarify the importance of leadership in any organized change. The way teachers act and represent their reality is strongly influenced by the architecture of their organization, while their ability to act with agency is directly linked to the existence of flat or prominent hierarchies, both potentially problematic for deep and systemic change. A hierarchical imposition from above as well as a lack of leadership vision in fragmented school cultures cannot determine any transformation.

In recent years, transformation has emerged as a high priority in key policy documents (OECD, 2015, 2020a, 2020b; Paterson et al., 2018; UNESCO, 2021) and been recognized as a major pillar on which the very future of education is based. A galvanized international scene has put transformation at the top of the agenda. One reason is found in the recent Covid-19 emergency and the need to recover, and possibly to “build back better”. Other reasons are longer-term and relate to dissatisfaction with the quality of education in many parts of the world. Major international agencies have been directly involved in reform and have variously endorsed “educational planning” (e.g., Carron et al., 2010), systemic reform in highly centralized countries, school autonomy (framed as school-based management or decentralization), systemic adjustment and restructuring (e.g., Carnoy, 1998; Samoff, 1999), and accountability (Anderson, 2005), as well as capacity building and development (De Grauwe, 2009). However, in practice, only segments of reforms have been enacted, focusing on one aspect of the school system while neglecting others, without considering the larger governance and school architecture, and local pedagogical cultures. Some agencies have also expressed a renewed interest in innovation and the possibility to measure it (Vincent-Lancrin et al., 2019), from a rather managerial perspective.

The transformation of education is a trendy movement nowadays, with the potential to generate lasting change through wide-reaching actions, not just stylistically or in local projects. Transformation of this kind will occur when structural and organizational conditions are in place in a range of different settings. When this happens, transformation as a revamped concept of change can be wholeheartedly embraced. Nonetheless, both academic and development-oriented NGO research has long dedicated itself to and learned from systemic change, improvement, and reform, based on what have been defined as effective practices (Ko & Sammons, 2016; Townsend, 2007). The school effectiveness findings are typically transversal principles of what has proved valuable despite contextual variation, whilst noting the local variability of such principles (Teddlie & Stringfield, 2017) especially in low and middle income countries (Moore, 2022) and even in similar areas of education development (Boonen et al., 2013; Palardy & Rumberger, 2008). Some variability often occurs between consolidated and less consolidated school systems. School improvement has been based on scholars’ findings on school effectiveness, as these two areas can merge up to a certain point (Creemers & Reezigt, 2005; Stoll & Fink, 1996). Reform at the top and improvement at the ground level have long been trialed in different national and organizational settings and with different school populations, with the aim of establishing generalizability or local variation. Quality teaching (Bowe & Gore, 2016; Darling-Hammond, 2021; Hattie, 2009) or teachers (Hanushek, 2010, 2014; Mincu, 2015; Akiba & LeTendre, 2017), as well as equitable effective practices (Sammons, 2010) have also been classic research topics that have emerged center-stage in any change project.

In order for quality-promoting endeavors such as change, improvement, and reform to produce a transformed education, several assumptions are indispensable: (a) recognize the larger school and organizational context as crucial, alongside school architecture and processes, (b) define what quality education means across a variety of country contexts and with regard to specific structural arrangements and pedagogical cultures, (c) distinguish the degree and type of autonomy for schools and teachers, and estimate the effectiveness of their mixed interactions, (d) understand and cope from a change perspective within a variety of school cultures, (e) recognize the structural limitations faced by school leadership, as well as the margins to produce local, gradual improvement that can pave the way to radical transformation, and (f) start any significant change at the school level, in the interaction of leaders and teachers.

What is school leadership and how can it bring about change? On the one hand, leadership is about a vision of change, collectively shaped and supported. In this sense, radical change—i.e., transformation—cannot occur without leaders and especially school leaders. In addition, an effective vision about a desired change grows from the interactions of the school actors and is stimulated and orchestrated by the school leadership. An imposition from above as well as a lack of leadership vision in fragmented school cultures cannot determine any transformation, nor its subsequent stability or growth, given that some grass roots changes happen accidentally, in limited school areas. In fact, if change is to be systemic and transformative, it cannot occur at the individual teachers’ level, as then it cannot be circulated and consolidated in stable, coherent collective practices. Action at the school level is fundamental for change to occur and last, as well as for individual teachers to be encouraged, supported, and rewarded for their innovative behavior. On the other hand, change is often conceptualized as a gradual process of a series of stages (Fullan, 2015; Kotter, 2012), carefully incorporating structural and cultural adjustments (Kools & Stoll, 2016). Transformation, a less orthodox and robust concept, incorporates the desire for more abrupt and radical change. It is imagined as a possibility to “leapfrog”. This desire to move rapidly forward resonates with the “window of opportunity” phase when big changes can occur more smoothly. However, at the school and even systemic level, complex changes resulting in net improvements are most often gradually prepared and stimulated, since any change is cultural in essence, and as such it needs time to occur. Another relevant aspect is related to leadership as an ingredient and quality, not just a positionality attribute. Both assumptions suggest the inevitability of its role to any change in education as an organized endeavor.

## Larger contexts and school organizations are key in any transformation

Education does not occur in an organizational vacuum, since deschooling, mass home-schooling, or online-only paradigms are neither implemented nor envisioned. In addition, a concept of education exclusively posed in philosophical and theoretical terms, especially when aimed at transforming the status quo, neglects to take into account that schooling is enmeshed with different organizational and governance forms, at times in contradiction with its own theoretical bases. Most important, forms of sociality such as those sustained by schools have not declined in relevance but increased, in the aftermath of the global online experiment of the pandemic emergency. At the same time, improvements and even radical changes in education have been embraced and actively promoted in certain parts of the world. For instance, in Norway, renewed weekly timetables are in place, allowing for deep learning as well as better integration with virtual knowledge in high-stakes exams. One should not forget that most pupils around the world are educated in environments displaying significant structural convergences across countries, despite locally diverse values. Such teaching-oriented settings are characterized by the centrality of the adult as teacher, and most often by textbook-based education. The organizational arrangements are linear, based on daily subjects and teachers’ contractual time, mainly dedicated to teaching activities (the *stavka* system, see Steiner-Khamsi, 2016, 2020) or to ad hoc self-help actions in extreme emergency contexts. Linked to these, school cultures can be both hierarchical (rules are delivered “from above”) and fragmented, since class teachers may be left to themselves without adequate professional support. Whilst the reality is nuanced and school typologies are in any case sociological abstractions, most systems can still be described as basically centralized or decentralized, depending on the level of autonomy granted to schools or local authorities. The larger school contexts as well as the local ones are even today very diverse in these two cases, despite a global increase in diversified combinations of centralization of some aspects and decentralization of others. What Archer (1979) theorized in her landmark work is still a key valid explanation of how school organizations usually operate and change. With renewed categories, a centralized system is largely characterized by “hierarchies”, real or perceived, and less by “networks and markets”, whilst in the case of decentralized systems, the opposite is true. The same differences can be highlighted in more comprehensive or selective school types, whose visions and ways of functioning are coherent with their structural patterns and influence, and in turn, with how leaders perceive their role and mission.

In terms of leadership, differing configurations will bring differing consequences. Centralized countries with weak school autonomy approach the role of school leaders in a rather formalist way: as *primus inter pares* or as administrative and legal head. In these settings, the intermediate level is also very weak and largely based on ad hoc tasks. Flat organizations may not support leadership as an essential element in the school’s operational life, and instead focus primarily on teaching, which is mainly viewed as an individual endeavor. School organizations at odds with leadership as a system quality, both in organizational and instructional terms, often exhibit forms of fragmentation (Mincu & Romiti, 2022), even in societies that may share a collectivistic or communitarian ethos, such as in East Asia. In countries with significant school autonomy, leadership structures are more manifestly in place, given the increased tasks performed by schools. Often, an excess of hierarchical leadership is a major negative outcome. However, the school context can be characterized by mixed combinations of types of governance (hierarchies, networks, markets) (Mincu & Davies, 2019; Mincu & Liu, 2022), which have a significant influence on the way leadership is oriented and how it accomplishes its visionary, organizational, and instructional functions within the school and in relation to society. School leadership is both a processual quality and a positional trait, and thus it can be variously performed in high autonomy school systems. In the case of centralized arrangements, it can be much harder to identify leadership as process where there is just some form of leadership positionality: a legal school head or the existence of subject-matter departments. School contexts and organizations around the world are also diverse in terms of leadership configurations and roles: some schools may share the same leader (Italy), some may not provide many leadership positions at all (India), and others may specify a headship position which does not in fact offer any leadership or cohesion in organizational and pedagogical matters. Indeed, leadership may be entirely missing from certain school systems.

To summarize, the way teachers act and represent their reality is strongly influenced by the architecture of their organization, along with the quality, direction, and margins of power that can be exerted by leadership at the school and intermediate levels. Nevertheless, schools are large organizations, and as such a certain amount of alignment and direction is needed, which is what leadership provides.

## The autonomy of schools and that of teachers are not mutually exclusive

Closely related to the first assumption, for a functional and dynamic school organization, a certain amount of school autonomy is required to adequately balance teachers’ autonomy. In high school autonomy systems, there is a tendency to assume that teachers’ autonomy is quite reduced, and this is certainly the case if the education model is accountability-oriented and leadership is hierarchical. In less autonomous systems, huge resistance to instill more autonomy at the school level is usually deployed—for example, in strongly unionist cultures, which aim to extend and expand teachers’ independence. This translates into quite radical teachers’ autonomy on pedagogical matters, as is the case in certain European school systems (Mincu & Granata, 2021).

An excess of teachers’ autonomy is detrimental to coherence and alignment at the school level and affects both quality and equity. The metaphors of teachers in their classes as eggs in their egg crates or lions behind closed doors, in the words of a ministry official in Italy, are particularly telling about flat, non-collaborative structures. The idea that high teacher autonomy may automatically support collegiality in flat organizations is not supported by the reality on the ground in certain school systems. In sociological terms, any human organization requires a certain amount of hierarchy and collegiality. In fact, a certain quantity of school autonomy is beneficial in many ways and can enhance teachers’ agency: (a) it emphasizes the role of leaders, including the possibility for teachers to act with leadership, (b) it offers a direction that can be shared, (c) it stimulates people to come together in effective ways (communities of practice) whilst presenting the risk of some contrived collegiality, and (d) it encourages teachers to feel more supported in their own work and professional development.

In a nutshell, leadership’s margins of influence are shaped not only by overall system governance, but also by the amount of school autonomy they enjoy. In addition, the extent of organizational autonomy is directly linked to the existence of flat or prominent hierarchies, both potentially problematic for deep and systemic change.

## School cultures converge and diverge in multiple ways within and across countries

Pedagogical transformation is about a change in cultural assumptions, which entails a slow process of cognitive and emotional modification that has to be supported beyond school walls by concerted social and economic actions. Structural change will not be successful without an adjustment in people’s cognitive schemes about their practices and values. How teachers conceive of teaching and learning, and of equitable and inclusive approaches, is not essentially a matter of “lack of training”, for which more preparation may be the solution. It is instead a matter of deep pedagogical beliefs, whose roots are shared and societal. How to discipline class misbehavior, for example, and even what inappropriate classroom behavior is, varies widely across societies: it denotes (generational at times) power distance, gender relations, assumptions about individuality and collectivistic entities, as well as merit recognition and social envy avoidance. For Hargreaves (1994), school culture is the result of the intertwining of attitudes such as individualism, collaboration, contrived collegiality, and “balkanization”, i.e., fragmentation of ethical goals. Stoll (2000) herself describes schools in terms of social cohesion and social control as traditional, welfarist, “hothouse”, or anomic. In contrast, for Hood (1998), there are four possible combinations of social cohesion and regulation: (a) fatalistic: compliance with rules but little cooperation to achieve results, (b) hierarchical (bureaucratic): social cohesion and cooperation and a rules-based approach, (c) individualist: fragmented approaches to organizing that require negotiation among various actors, and (d) egalitarian: very meaningful participation structures, highly participatory decision-making, a culture of peer support.

In reality, mixed combinations of two, three, or more types of cultures can be found and supported by a variety of factors within and beyond schools as organizations. Some Southern European realities, as well as some Eastern European systems, belong to the individualist typology: weak collaboration and weak hierarchy, given the absence of a teaching career structure with levels of preparation and strong autonomy of the individual teacher. Some aspects of institutional “fatalism” are present, because a certain culture of respect for rules nevertheless exists, and of egalitarianism of a rather formal type. In fact, while the collegial culture on a formal level may appear robust—given the presence of collegial bodies—in practice organizational coherence remains very weak. The reason lies in the fact that these bodies can also decide not to agree on any systemic solution and defer decisions to the individual teacher, since teacher autonomy is still the superior criterion governing informal culture in schools. In the Anglo-Saxon and Scandinavian school systems, for example, schools express more coherent and cohesive cultures that oscillate between very hierarchical and more participatory models, with more diffuse leadership (Seashore-Louis, 2015). Even though these latter school systems favor a mostly cohesive ethos, it is not uncommon to find fragmented and inconsistent schools with weak leadership.

As an example of how school cultures work, a culturally well-rooted premise that teachers “are all good” is very much at work in certain flat hierarchical or Confucian-oriented school cultures, meaning they are equally effective because morally oriented for the profession. This is, in fact, a convenient belief allowing those within it to oppose forms of evaluations (including between peers and in the wider community of parents and stakeholders) and to resist more school autonomy and cohesiveness measures that might be envisioned by school or system leadership. Whilst teachers may be reluctant to work together and observe each other (as in a lesson study format) in most countries, this may be particularly the case where teachers’ autonomy is quite radical, where collaboration and mentoring are not common practices, or where stimulated by school arrangements and work contracts (e.g., in Italy; see Mincu & Granata, 2021).

Another way to characterize pedagogical cultures is with reference to formalism (respect for rules and social distances, focus on adults’ role and transmissive pedagogies) or to progressivism (more egalitarian interactions and a focus on the learner and their way of acquiring and creating knowledge). There are many ways in which various school cultures can be appropriately characterized, offering plenty of nuances and details of social, economic, and cultural stratifications and contradictions: for instance, in certain East Asian contexts, there is a combination of Confucianism, socialist egalitarianism, and revised individualism of consumption or of possession, based on previous rural forms of it. However, along the lines of centralized/decentralized typologies that are still valid for describing school functioning and structures, the reality of countries around the world allows scholars to characterize school cultures as formalist versus progressivist. It is legitimate to do so in spite of the local nuances and anthropological cultures that may filter and support such pedagogies (Guthrie et al., 2015).

Any cultural change imposed from above or from abroad may be doomed to failure if the hardware is that of centralized systems and if school actors are not allowed to engage in a cultural exercise of adaptation, adequately supported with infrastructural measures. Whilst there is no single model, there are some pillars of good teaching and some key lessons about how to produce change. A major premise is that any change must reach the school level and be able to activate and energize its school actors. School systems may be distinguished therefore in terms of formalist/progressivist typologies, which is coherent with other types of systemic characteristics, including lack of leadership (be it hierarchically formalized, legally representative only, or peer-oriented) that may preclude any effort of cultural transformation.

Without leadership, individual teachers may act as a loosely connected group, without vision and motivation to produce an expected and socially praised change. The expectation to encourage reforms from the regional and district level, when not from the top, is purely utopian. Schools remain remote realities in such change models. Most systems in poorly resourced contexts are entangled in hierarchical school models and grounded in traditional power distance and colonial legacies. Without significant leadership processes stimulated by school principals at the very heart of such systems, cultural and new structural processes cannot be expected. To produce cultural change, the top leadership stratum must create the proper conditions, such as salaries, workload, and other incentives for training and knowledge dissemination; *but action and cognitive schemes characterize the school level and teachers cannot be blamed for what they cannot do by themselves.*

## Defining quality for present times education in context

We cannot move toward possible futures without deeply understanding what good education can be in our present societies, in a variety of localities around the world. Research has long dedicated itself to the task of defining quality in education, particularly in the fields of school effectiveness and school improvement. Meta-research has become a bestseller scholarly genre (Hattie, 2009), and the drive toward evidence-based knowledge has been equally impressive, across universities, NGOs, and other major international players. Research studies distinguish between quality teachers (their attributes, amount of preparation, and years of experience) and teaching quality, based on dimensions of quality teaching that produce effective learning. Since structures and cultures can be effectively encapsulated in categories (centralized/autonomous, formalist/progressivist, etc.), quality teaching is also condensed (a) in key dimensions, for instance by Bowe and Gore (2016), subsuming further aspects, or (b) as rankings of most effective factors in terms of learning.

Mistrust of evidence-based and best-practice research traditions is justified when ready-made solutions are implemented without adaptations and the engagement of those involved. Even the adoption of South-South solutions can be ineffective at times (Chisholm & Steiner-Khamsi, 2008). Since problems in education are messy and “wicked” (Ritter & Webber, 1973) changes must be systemic and cultural.

Anderson and Mundy, 2014 proved that improvement solutions and practices in two groups of countries—developed and less developed—are very much convergent. Both developing and developed countries present a series of common challenges: the need for fewer top-down approaches, for instance, and for approaches less narrowly focused on the basics. Comparative evidence and perspectives on student learning in developing countries converge on a common cluster of instructional concepts and strategies: (a) learning as student-centered, differentiated, or personalized, associated with using low-cost teaching and learning materials in the language which students understand, and (b) the appropriate use of small group learning in addition to large group instruction. This enables regular diagnostic and formative assessment of student progress to guide instructional decision-making, clear directions, and checking student understanding of the purpose of learning activities. It also involves personalized feedback to students based on assessments of their learning, and explicit teaching of learning skills to strengthen students’ problem-solving competencies. With the possible exception of low-cost learning materials, these prescriptions for good teaching are consistent with international evidence about effective instruction (Anderson & Mundy, 2014). But quality teaching and teachers equally assume specific contextual meanings. For instance, Kumar and Wiseman (2021) indicate that traditional measures of quality (teacher preparation and credentials) are less relevant in India compared to non-traditional measures such as teachers’ absenteeism and their attitude/behavior toward their students.

## Teachers alone cannot make a better school

Teachers and their actions at the classroom level are key to inspiring learning and students’ progress. Nonetheless, a misreported finding from an OECD (2010) study that “the quality of an education system can never exceed the quality of its teachers” is only partially correct. In fact, the full quotation said that the system’s quality cannot exceed the quality of its teachers and leaders. The incomplete quote mirrors a common misconception that teachers alone can and should improve the system. Instead, teachers are part of organizations, and as such they behave and respond to dynamics in place in those contexts, and not as individuals, or as a professional group, not even in the most unionized countries. The quality of a public service cannot be attributed solely to its members, but also to their organization and to specific choices made by its leadership, which is responsible for organizational vision and translating theories into action. Launching heartfelt calls for teachers to change their practices is both naive and sociologically inaccurate regarding how people act and behave in social organizations, such as schools. The presence of leadership as a processual and qualitative dimension at the school level also indicates the existence of the structures of school leadership teams and middle managers, in which leadership is robustly in place as positionality.

In this sense, the quote indicates the relevance of teachers’ work in carefully designed organizations, in which hierarchy and horizontal interactions of collaboration between peers are in a functional equilibrium. In other words, schools and teachers’ autonomy reciprocally reinforce one another.

Whenever teachers are required to act with leadership, autonomy, and innovation, the larger system and school culture should be carefully considered. Teachers cannot by themselves be directly responsible for systemic changes. National-level teams of experts cannot blame teachers for a lack of change when the necessary knowledge and resources are not cascaded effectively to the school level. As the end point of the chain of change, teachers cannot be accused for a lack of success and adequate culture to facilitate innovation when decision makers do not consider the school architecture and how leaders are prepared and ready to support a change in culture. This has been the case with reforms in less resourceful countries around the world, often in highly centralized systems, where more progressivist changes are expected from teachers in the absence of proper consideration of the school architecture, long-standing interactions with the school leaders, and the overall pedagogical culture. Unfair blame for these teachers is expressed at times by international or national teams of experts, unrealistically expecting individual teachers to produce significant structural and cultural changes, otherwise they play the part of “those who wait on a bus” for a change to happen. The possibility to develop, to act innovatively, and to be motivated for teaching depends largely on the organizational support received by teachers at the school level from their head teacher and the wider environment. Professional development is a key ingredient that impacts teacher quality (Cordingley, 2015), and its effectiveness and provision depends heavily on the school leadership. Without support from the larger school context and leadership, even the most autonomous teachers may not act with the necessary teaching quality that can make a difference, as clearly illustrated by TALIS 2020.

## Leadership, as an organizational quality, is indispensable

The final assumption involves the idea that one cannot crudely distinguish between teachers and leaders, especially middle managers and more informal leaders. Obviously, there is a continuum between such roles: teachers themselves can act with agency and leadership, formally or informally, and head teachers may draw upon their experience as teachers.

Since schools are organizations and not collections of individuals, the field of school effectiveness and school improvement has incontrovertibly identified the influence of leadership as vital: “school leadership is second only to classroom teaching as an influence on pupil learning” (Leithwood et al., 2008). Through both organization and instructional vision (Day et al., 2016), effective leadership significantly enhances or diminishes the influence that individual teachers have in their classes. Regardless of cultural considerations, when teachers’ work is uncoordinated and fragmented, the overall effect in terms of learning and education cannot be amplified and adequately supported. A lack of coherence within organizations is unfavorable to more localized virtuous dynamics that may be diminished or suffocated.

Moreover, unjustified allegations of managerialism and the striking absence of this topic from key policy documents, including those of UNESCO (2021), should be highlighted. Whilst the “executive” components implicit in any leadership function must be in place in organizations enjoying wide autonomy, this does not necessarily translate into managerialism and quasi markets. It is indeed the larger school context that can make an autonomous school perform in a managerial way or simply, with broader margins of action, that can facilitate good use of teachers’ collective agency, as in some Scandinavian countries. In order to produce even modest change, let alone radical transformation, we must overcome the widely held misconception that leadership has to do with managerial tasks, competition, and effectiveness from a highly individualistic stance. Whilst this can be the case in certain country contexts and with particular disciplinary approaches, educational leadership does not simply overlap with managerialism as a technical ability. It is essentially about vision and collaboration around our global commons, as well as locally defined school goals.

School leadership is correctly identified as a key strategy to improve teaching and learning toward SDG4 (the Incheon Declaration and Framework for Action adopted by the World Education Forum 2015). A specific task assigned to school leadership is an increase in the supply of qualified teachers (UNESCO, 2016). At the same time, the need to transform schools is sometimes decoupled from the potential of school and system leadership to ensure such transformation. Failing to recognize the role of leaders in quality and equitable schooling must be rectified. A humanistic vision and a focus on the global public good cannot be at odds, programmatically, with a field dedicated to understanding how contemporary schools are organized and how they operate.

## Conclusion: Leadership is about organized agency, not managerialism

Innovations in education are complex because they can often be incremental and less frequently radical, but some have the potential to be truly transformative. The more effective tend to be small micro-context innovations that diffuse “laterally” through networks of professionals and organizations but need facilitation and effective communication from above to be deep and long-lasting. They are never just technical or structural, but rather cultural and related to visions about education. In this context, leadership and leaders are crucial in a variety of aspects, but foremost in shaping a coherent organization and engaging collectively to clarify and make explicit key pedagogical and equity assumptions, which has a dramatic direct and indirect influence on the effectiveness of the school. Most significantly, school leadership at all levels is the starting point for the transformation of low-performing (and) disadvantaged schools.

We should not underestimate the impact that the larger political, social, and economic context has on schools and leaders around the world. A variety of autonomous schools can perform in a managerial way or simply make good use of teachers’ collective agency, and a variety of less autonomous organizations may dispose or not of a certain dose of organizational coherence and leadership (Keddie et al., 2022; Walker & Qian, 2020).

What has proved valuable in most contexts may not always be effective in every case; a balance has to be struck between cultural awareness related to pedagogies in contexts and lessons learned across cultural boundaries. Available universal solutions have to be pondered, and adaptations are always required. It can be the case that, in certain conditions, we borrow not only solutions but the problems they address, in the way these are rhetorically framed. However, since convergences occur in structures and cultures, problems may also converge across contexts. In addition, micro-changes occur fluidly at any time, but for transformation to emerge, we need to draw on the accumulated wisdom and the potential implicit in system and school leadership. Last but not least, the complexity lying at the heart of learning from others and from comparison should not be assumed to be insuperable.
